# Examination of the relationship between thanatophobia and resilience levels of nurses working in intensive care and palliative care units

**DOI:** 10.1186/s12912-023-01405-7

**Published:** 2023-08-24

**Authors:** Mahruk Rashidi, Funda Karaman, Gülay Yildirim, Neşe Kiskaç, Gülşah Ünsal jafarov, Buse Saygin şahin

**Affiliations:** 1 Faculty of Health Sciences, Department of Nursing, Istanbul Gelisim University, Istanbul, Turkey; 2 Department of Nursing, Trakya University, Kesan Hakki Yoruk School of Health, Edirne, Turkey

**Keywords:** Nurse, Intensive care, Palliative care, Thanatophobia, Psychological resilience

## Abstract

**Background:**

Nurses in critical care and palliative care units care for patients suffering from severe pain and suffering and at high mortality risk. For this reason, nurses working in these units should be psychologically resilient. However, nurses who are constantly exposed to the death process face the risk of thanatophobia. The aim of this study is to examine the relationship between thanatophobia levels and the psychological resilience of nurses working in intensive care and palliative care units.

**Methods:**

The sample of this descriptive and cross-sectional study included 158 nurses working in intensive care and palliative care units. Personal information form for nurses, Thanatophobia Scale and Psychological Resilience Scale for Adults were used. Data were collected through an online questionnaire in the study. Percentage calculations, mean measurements, Kruskal Wallis test and Mann Whitney U test were used in the statistical evaluation of the data.

**Results:**

The mean of thanatophobia scale and psychological resilience scale was found 31.74 ± 10.08 and 108.34 ± 7.12, respectively. There was a statistically significant difference between the tanatophobia total scale score and age, receiving training on psychological resilience (p < 0.05). A statistically significant difference was found between perseption of self, family cohesion and perception of future and the status of receiving training on psychological resilience (p < 0.05). A statistically negative significant correlation was determined between the thanatophobia scale and the psychological resilience scale total scores.

**Conclusions:**

As a result, it was determined that as the thanatophobia of the nurses increased, their psychological resilience decreased. This situation may negatively affect nurses working in critical departments to provide quality health care to patients. Establishing and maintaining training programs to reduce thanatophobia and increase psychological resilience of nurses working in intensive care and palliative care units will ensure that nurses provide quality health care to the patient and reduce the physiological and psychological wear of nurses.

## Introduction

Death is a universal event experienced by all living organisms, and it is the last stage in the psychological and physical sense, in which the vital tasks of living things cease to be repeated [1]. In addition to being a biological phenomenon, death is an indistinguishable part of lives, and as a result of the differences that people create with themselves and their environment, people’s perception of death varies from person to person [2]. In recent years, medical and technological developments that prevent the end of life have turned death from being a personal and spiritual event into a medical event that needs to be fought [3].

Intensive care and palliative care units are areas where human life is at the limit and patients who require care by a professional team are accepted. Nurses working in these units face more patient loss compared to nurses work in other units. Both inpatients in intensive care and palliative care units and healthcare professionals caring for them are faced with the risk of thanatophobia. Thanatophobia is the fear of one’s own death or the process of death. Almost every human being fear death and this fear differs from any other fear as it is very strong [4, 5].

Nurses in palliative care and intensive care units, witnessing high mortality rates, go through significant fear of death, and may be unable to cope with it, and thus become physiologically and psychologically worn out [6]. Nurses, in particular, spending more time than other healthcare professionals with terminal patients who are in significant pain and suffering, may begin to think that they failed in the care they gave and feel professionally inadequate or guilty [7]. The psychological resilience levels of nurses are also affected by the difficulties they experience in their professional lives. Psychological resilience is the ability of people to recover or overcome various difficulties and challenges they encounter in life [8]. Thus, nurses need to be psychologically resilient in order not to wear out professionally and personally [9, 10].

### Literature review

Kösedağ (2021) determined that 150 intensive care nurses experienced intense fear of death. Almegewly et al. (2022) found that 139 intensive care nurses had high levels of stress and anxiety during the COVID-19 outbreak. Studies show that nurses working in intensive care units experience stress, anxiety and fear of death [11–13].

Han et al. (2022) conducted a qualitative study with intensive care nurses during the COVID-19 pandemic and found that nurses had serious emotional reactions, both physical and emotional, and needed support related to psychological resilience from multiple sources [14]. Hasani et al. (2022) looked at the effect of resilience training on job stress in intensive care nurses and found that their job stress was reduced after training compared to the control group [15]. Leppin et al. (2014) found that stress-oriented training programs were effective in increasing psychological resilience [16].

There is no study in the literature examining the thanatophobia and psychological resilience levels of nurses in intensive care and palliative care units. The aim of this study is to examine the relationship between the levels of thanatophobia and resilience of nurses working in intensive care and palliative care units. The result of the study will contribute to the training programs that can be developed for nurses working in these units.

The research questions are as follows;

- What are the thanatophobia levels of nurses working in intensive care and palliative care units?
- What are the psychological resilience levels of nurses working in intensive care and palliative care units?
- What is the relationship between the tanatophobia levels and psychological resilience of nurses working in intensive care and palliative care units?

## Methods

### Research aim and design

This study was conducted as a descriptive and cross-sectional study to determine the relationship between the thanatophobia levels and psychological resilience of nurses working in intensive care and palliative services.

### Sample, place and time of the research

The population of the study consisted of 390 nurses who met criteria and working in the intensive care and palliative units of the city hospitals in Istanbul, where the number of intensive care and palliative unit beds and patient diversity are high. The research sample was determined as 151 nurses according to the known population sample calculation with margin of error of 5% with a 95% confidence interval. All 158 nurses who agreed to participate in the study and met the criteria were included in the sample. The inclusion criteria of the participants were to have worked in the intensive care and palliative unit for at least 6 months and to agree to participate in the study. Research data were collected between November 2022 and January 2023.

### Data collection and data collection tools

The research data were collected using personal information form prepared by the researchers, the Thanatophobia Scale, and the Psychological Resilience Scale for Adults. The data were obtained from Google Forms application sent by the researchers to participant’s e-mail. The response rate to the tools used in the study was 41%.

#### Personal information form

The form has 7 questions on nurses’ personel characteristics (age, gender, marital status, the high school they graduated from, whether they have children, which unit they work in, and their education level on psychological resilience).

#### Thanatophobia scale

The scale was first developed in 1998 by Merrill et al. [17]. Turkish validity and reliability studies of the scale were conducted by Çiftçioğlu and Seren in 2019 [18]. The 7-point Likert-type scale includes seven items in total with the items scored as “strongly disagree” through “strongly agree”. The Cronbach’s alpha value of the Thanatophobia (Fear of Death) Scale was 0.92 in the original study, 0.96 in the Turkish version, and 0.87 in our study. As the average score obtained from the scale increases, it is evaluated and interpreted as the person’s fear of death increases. The lowest score that can be obtained from the scale is 7, and the highest score is 49.

#### Psychological resilience scale for adults-PRSA

The scale was developed by Friborg et al. and was translated into Turkish and verified for validity and reliability by Basım and Çetin [19, 20]. It contains 33 items and 6 subscales, which are ‘structural style’ (3,9,15,21), ‘perception of future’ (2,8,14,20), ‘family cohesion’ (5,11,17,23,26,32), ‘perception of self’ (1,7,13,19,28,31), ‘social competence’ (4,10,16,22,25,29) and ‘social resources’ (6,12,18,24,27,30,33).

If increased scores are to indicate increased resilience, the answer boxes should be rated from 1 to 5 from left to right. In this option, questions 1–3–4–8–11–12–13–14–15–16–23–24–25–27–31–33 will be reverse questions (if decreased scores are to indicate increased resilience, the answer boxes should be rated from 5 to 1 and reverse items would then be questions 2–5–6–7–9–10–17–18–19–20–21–22–26–28–29–30–32). The first option was chosen in this study. The total Cronbach’s alpha coefficient of the original scale was 0.86, and it was 0.90 in this study. The lowest score that can be obtained from the scale is 33 and the highest score is 165. An increase in the scores obtained from the scale means that the psychological resilience levels of the participants are high.

### Data analysis

The data were analyzed by using IBM *SPSS Statistics 21.0.* The test to identify whether the data showed normal distribution demonstrated non-normal distribution and Mann Whitney U and Kruskal Wallis tests were therefore used. Descriptive statistical methods such as frequency, arithmetic mean, standard deviation, and percentage were used to examine the descriptive characteristics of the students, and Spearman Correlation analysis was used to determine the relationship between the scales.

## Results

39.2% of the nurses were in the 20–25 years age range, 86.1% were women and 62% were single. Most had a bachelor’s degree (72.8%) and no children (75.3%). 56.3% were working in intensive care units and 93% did not receive training on psychological resilience (Table 1).

**Table 1:** Personal Characteristics of Nurses (n = 158)

Personal Characteristics		n	%
**Age**	Age 20–25 years Age 26–35 years 36 years and above	62 58 38	39.2 36.7 24.1
**Gender**	Female Male	136 22	86.1 13.9
**Marital Status**	Married Single	60 98	38.0 62.0
**Education**	High School/Associate Degree Bachelor’s degree Postgraduate degree	24 115 19	15.2 72.8 12.0
**Has children**	Yes No	39 119	24.7 75.3
**Unit they work in**	Intensive Care Palliative	89 69	56.3 43.7
**Status of Receiving Training on Psychological Resilience**	Yes No	11 147	93.0 7.0

Thanatophobia Scale score of nurses were found as given in Table 2. The mean score of the Thanatophobia scale of the nurses was found to be above the medium level.

**Table 2:** The Thanatophobia Scale Scores of the Nurses (n = 158)

Minimum Maximum			Mean ± SD
7	49		31.74 ± 10.08

When the total score of the tanatophobia scale was compared according to the personal characteristics of the nurses; While there was no statistically significant relationship between the tanatophobia total scale score and gender, marital status, having a child, and the unit in which she worked (p > 0.05), there was a statistically significant difference between the tanatophobia total scale score and age, training in resilience (p < 0.05).Those whose age range is between 20 and 25 and those who had not received training on psychological resilience have a higher total scale score for Thanatophobia.

Mean score of PRSA and subscale scores are given in Table 3. The psychological resilience levels of the participants were middle level.

**Table 3:** Mean Scores of PRSA and Subscales (n = 158)

Scale Total and Subscales	Mean score (Mean ± SD)
Scale Score Total	108.34 ± 7.12
Structural style	13.62 ± 3.34
Perception of future	12.88 ± 2.02
Family Cohesion	18.83 ± 2.52
Perception of self	34.56 ± 3.78
Social competence	18.17 ± 11.73
Social resources	23.15 ± 2.34

When the mean scores of the PRSA and its sub-scales for adults were compared according to the personal characteristics of the nurses; There was no statistically significant relationship between age, gender, unit of employment, marital status, having a child, and total scale score and all sub-scales (p > 0.05).

There was no statistically significant relationship between the status of receiving training in psychological resilience and the total scale and structural style subscale, social competence subscale, social resources subscale (p > 0.05). A statistically significant difference was found between perseption of self, family cohesion and perception of future and the status of receiving training in psychological resilience (p < 0.05). It was determined that those who were trained in resilience had higher mean scores of perception of selfi, family cohesion and perception of future sub-scales.

There was a statistically significant negative correlation between the Thanatophobia Scale total and PRSA scale mean score (p = 0.00).

## Discussion

Nurses working in palliative care and intensive care units face too many deaths and have fear of death. This causes physiological and psychological wear and can also affect the psychological resilience of nurses. The aim of this study was to examine the relationship between the levels of thanatophobia and resilience of nurses working in intensive care and palliative care units.

The Thanatophobia Scale mean score of nurses was found to be above the medium level at 31.74 ± 10.08 in this study. There are studies examining the thanatophobia levels of nurses and reported moderate to high levels [11, 20–22]. In a qualitative study by De Swardt and Fouche (2017), it was determined that nurses who gave post-mortem care to the deceased in the intensive care unit experienced thanatophobia by confronting the reality of their own death [23]. In a study conducted with trainee nurses in China, the trainee nurses’ attitude scores towards death were at a moderate level [24]. Studies show that nurses experience thanatophobia and have moderate or high levels of thanatophobia. The results of this study were similar to other studies [11, 20, 24].

Thanatophobia mean scores of those aged 20–25 were higher in our study (p < 0.05). A study examining nurses’ knowledge of palliative care and attitudes towards the care of dying patients found that as the age of nurses increased, their thanatophobia scores decreased and they had more positive attitudes towards death [25]. Another study found that as the age of nurses increased, their avoidance of death decreased, and they accepted death as a natural phenomenon [26]. Nurses with more work experience were found to be more inclined to have more positive attitudes towards death [26–28]. It is interpreted that younger nurses aged 20 to 25 years having higher thanatophobia levels could be because of their more negative attitudes towards death and less professional experience than older nurses.

Nurses who did not receive training on psychological resilience had higher thanatophobia scores in our study (p < 0.05). Wilson et al. (2016) found that thanatophobia scores decreased as palliative care knowledge levels increased. It was found that education on life, death and bereavement can improve attitudes towards the care of dying patients [29]. In Argentina, Spain and Italy, training given to medical school students on the care of dying patients was found to positively affect students’ attitudes towards dying patients [30]. Studies are mostly focused on how the attitude towards death is affected by the education given on palliative care, life, death, bereavement and the care of dying patients. Different training programs seem to be effective in reducing the thanatophobia of nurses. This research shows that training on resilience is also effective in reducing the level of thanatophobia of nurses.

In the study, the nurses’ Psychological Resilience Scale for Adults mean score was moderate at 108.34 ± 7.12. A study examining the effect of the psychological resilience of emergency nurses on their thanatophobic behaviours determined that nurses had moderate psychological resilience [22]. There are studies in the literature showing that nurses’ psychological flexibility of nurses working in different departmentsis moderate [31, 32]. Resent research is coherent with other studies in the literature.

Psychological resilience decreased as thanatophobia increased in our study (p = 0.00). Kartal et al., in their 2022 study with nurses working in the emergency room, emphasized that there was a moderately negative relationship between resilience and thanatophobia and that 40.9% of psychological flexibility was related to thanatophobia [22]. There are no studies examining the relationship between thanatophobia and psychological resilience in nurses working in intensive care and palliative care units. This study supports the fact that as the level of thanatophobia in nurses increases, psychological resilience decreases. Further studies on this topic are needed.

## Conclusion

As a result, the thaatophobia levels of the nurses were above moderate and their psychological resilience was moderate. Psychological resilience was found to decrease as thanatophobia increased. This situation may negatively affect nurses working in critical departments to provide quality health care to patients. Among the health policies, the patient has the right to receive quality care. For this reason, it can be recommended that nurses working in intensive care and palliative care units be given in-service trainings that teach effective coping methods to prevent fear of death and strengthen psychological resilience and ensure its continuity. This will ensure that nurses provide quality health care to the patient and reduce the physical, psychological and social wear of nurses.

### Limitations

The study is limited by several factors. The presented study was conducted in Istanbul Province and most of the participants were women. The results may be inconclusive when compared to the general population, since it was carried out in a group living in the same region and the participants were female. For this reason, it may be recommended to repeat the study in larger and widespread groups and to perform it in groups where the sample group is more homogeneous.

## Data Availability

The datasets used and/or analyzed during the current study are available from the corresponding author upon reasonable request.
